# Polyclonality of BRAF mutations in primary melanoma and the selection of mutant alleles during progression

**DOI:** 10.1038/sj.bjc.6606072

**Published:** 2011-01-11

**Authors:** J Lin, Y Goto, H Murata, K Sakaizawa, A Uchiyama, T Saida, M Takata

**Affiliations:** 1 Department of Dermatology, Shinshu University School of Medicine, Matsumoto, Japan; 2 Department of Dermatology, Okayama University Graduate School of Medical, Dentistry and Pharmaceutical Sciences, Okayama, Japan

**Keywords:** laser-capture microdissection, oncogene mutation, single-cell PCR, targeted therapy, V-raf murine sarcoma viral oncogene homologue B1

## Abstract

**Background:**

Oncogenic *BRAF* mutation had been considered to be a founder event in the formation of melanocytic tumours; however, we recently argued against this notion by showing marked polyclonality of BRAF mutations in acquired melanocytic nevi (Lin *et al*, J Natl Cancer Inst., 2009; 101:1423–7). Here, we tested whether similar heterogeneity of BRAF mutations exists in primary melanomas.

**Methods:**

We isolated and sequenced single melanoma cells from five primary melanoma tissues using antibodies against human high-molecular-weight melanoma-associated antigen. We also examined 10 primary melanomas by the sensitive Mutector assay detecting the BRAF^V600E^ mutation, as well as by cloning and sequencing of separated alleles. Furthermore, we estimated the frequency of BRAF mutant alleles in paired samples of primary tumour and recurrence or metastasis in three patients.

**Results:**

Single-cell mutation analyses revealed that four of five primary melanomas contained both BRAF-wild-type and BRAF-mutant tumour cells. Tumour heterogeneity in terms of BRAF mutations was also shown in 8 of 10 primary melanomas. Selection of BRAF mutant alleles during progression was demonstrated in all the three patients.

**Conclusion:**

Acquisition of a BRAF mutation is not a founder event, but may be one of the multiple clonal events in melanoma development, which is selected for during the progression.

Among the several genetic alterations governing melanoma initiation and progression so far identified, the serine/threonine-specific protein kinase BRAF is thought to be a key player because it is activated by somatic mutations in 50–70% of cutaneous melanomas. A substitution of glutamic acid for valine at codon 600 (V600E) is the most common BRAF mutation in melanoma, occurring in over 90% of BRAF-mutated cases (Davies *et al*, 2002). BRAF^V600E^ constitutively activates the RAS/RAF/MEK/ERK signalling pathway, and stimulates transformation of immortalised melanocytes (Wellbrock *et al*, 2004). Interestingly, the BRAF^V600E^ mutation was also found in over 80% of melanocytic nevi, suggesting that mutational activation of the RAS/RAF/MEK/ERK pathway is a critical step in the initiation of melanocytic neoplasia (Pollock *et al*, 2003). Moreover, a mouse model of melanocytic nevus and melanoma, which is driven by the inducible expression of BRAF^V600E^ in melanocytes, has been developed recently, providing further evidence that the acquisition of a BRAF mutation can be a founder event in melanocyte transformation (Dhomen *et al*, 2009). Based on these observations, a number of Raf kinase inhibitors, as well as a selective inhibitor of active B-Raf kinase have been developed and tested in clinical settings (reviewed in Gray-Schopfer *et al*, 2007; Wellbrock and Hurlstone, 2010).

Very recently, however, we have demonstrated marked polyclonality of BRAF mutations in acquired melanocytic nevi, which argues against the possibility that a mutation in BRAF is an initial event in melanocyte transformation (Lin *et al*, 2009). In this study, we have tested whether a similar heterogeneity of BRAF mutations exists in primary melanomas.

## Materials and methods

### Tissues

The study was approved by the medical ethics committee of the Shinshu University School of Medicine, and conducted according to the Declaration of Helsinki Principles. Primary and metastatic melanoma tissues were obtained from 17 patients. Case 17 was an autopsy case. For excisions, all patients gave written informed consent.

### Cell line

A melanoma cell line (MMG1) established from a primary acral melanoma was kindly provided by Professor Akifumi Yamamoto (Saitama Medical Collage Cancer Center, Saitama, Japan). The cells were maintained in RPMI 1640 medium containing 10% fetal bovine serum. Tyrosinase expression was confirmed by RT–PCR. This cell line was tumourigenic in NOD/SCID mice. Passages of >150 were used throughout this experiment. Although melanoma cell lines frequently show copy number increase at chromosome 7q where the BRAF gene resides (Tanami *et al*, 2004), array CGH analysis of MMG1 revealed no copy number aberrations at 7q (data not shown).

### Immunomagnetic cell isolation

Fresh tissues of five primary melanomas (PM-1–5) were used for isolation of pure melanoma cells by using an antibody cocktail of human high-molecular-weight melanoma-associated antigen (HMW-MAA) (a gift from Dr Soldano Ferrone, University of Pittsburgh Cancer Institute) and immunomagnetic beads, as described previously (Lin *et al*, 2009).

### Microdissection and DNA extraction

Primary melanoma cells separated by immunomagnetic beads and MMG1 cells were smeared on a film-coated glass slide (Maiwafosis Co. Ltd, Osaka, Japan), stained with methylene blue and subjected to laser-capture microdissection using a PALM-MB microdissection system (PALM Microlaser Technologies, Bernried, Germany). For the frozen tissues of primary and metastatic melanoma, 6-*μ*m-thick cryosections stained with methylene blue were manually microdissected on an inverted microscope to select the areas in which at least over 75% of cells were tumour cells. For formalin-fixed paraffin-embedded tissues, we carried out laser-capture microdissection to collect pure tumour cell populations with the aid of gp100 immunostaining using an antibody to gp100 (Zymed, San Francisco, CA, USA) and the DAKO Envision System (DAKO Japan Co. Ltd, Kyoto, Japan). DNA was extracted as described previously (Ichii-Nakato *et al*, 2006; Lin *et al*, 2009).

### PCR for BRAF exon 15

Procured single-melanoma cell samples were amplified for exon 15 of the BRAF gene with a hemi-nested PCR (Lin *et al*, 2009). DNA extracted from frozen or paraffin-embedded tissues was amplified by a conventional PCR (Ichii-Nakato *et al*, 2006). The PCR amplicons were purified using the QIAquick PCR purification kit (Qiagen Inc., Tokyo, Japan).

### Subcloning

The TA cloning was done with the TOPO XL PCR Cloning Kit (Invitrogen, Carlsbad, CA, USA) according to the manufacturer's instructions. After incubation at 37 °C overnight, colonies were picked up randomly from the plate, confirmed for correct recombination by PCR and inoculated into 3 ml of LB medium containing 50 *μ*g ml^−1^ kanamycin. After incubation at 37 °C overnight with vigorous shaking, the bacterial cells were harvested and purified using the QIAprep Spin Miniprep kit (Qiagen Inc.).

### Sequence analysis and identification of mutations

The purified PCR amplicons and recombinant plasmid DNA were sequenced using the BigDye Terminator v3.1 cycle sequencing kit (Applied Biosystems, Foster City, CA, USA) and analysed on an ABI PRISM 3100 Genetic Analyzer (Applied Biosystems).

### Mutector assay

DNA samples from primary melanomas were analysed for the BRAF^V600E^ mutation by a sensitive shifted termination assay using the Mutector kit (TrimGen Corporation, Sparks, MD, USA) following the manufacturer's instructions (Ichii-Nakato *et al*, 2006).

## Results

To investigate whether primary melanoma cells are homogenous or heterogeneous in terms of BRAF mutation, we first carried out immunomagnetic isolation of single melanoma cells from five fresh primary melanoma tissues using melanoma-specific human HMW-MAA monoclonal antibodies followed by PCR and sequencing, as previously described (Lin *et al*, 2009). High–molecular-weight melanoma-associated antigen is known to be expressed in >90% of melanomas, although the frequency is somewhat lower in acral melanomas (Campoli *et al*, 2004). Although HMW-MAA is also expressed in skin cells within hair follicles and epidermal basal cell layer, endothelial cells and pericytes (Campoli *et al*, 2004), we obtained virtually no cells from normal skin samples in our previous control experiments using the same cocktail of HMW-MAA-specific monoclonal antibodies (Lin *et al*, 2009). Single melanoma cells were selectively captured by immunomagnetic beads and procured by laser-capture microdissection (Supplementary Figure S1a). The tumours examined included three acral melanomas and two melanomas on non-chronically sun-damaged skin (Table 1). In four of the five primary melanomas, although the majority of the cells were BRAF-wild type, we observed a substantial number of melanoma cells harbouring the V600E mutation. Furthermore, two primary melanomas (cases 1 and 3) also contained tumour cells with other BRAF mutations, such as K601R, V600M and V600-K601E, all of which have been reported in melanoma (Garnett and Marais, 2004) (Supplementary Figure S1b). The V600-K601E mutations was shown to induce high BRAF kinase activity (Hou *et al*, 2007), whereas the activity of the K601R and V600M mutations have not been tested (Garnett and Marais, 2004). In one acral melanoma (case 5) all the separated melanoma cells were wild type. A primary melanoma cell line MMG1 also showed BRAF mutation heterogeneity (Table 1). Detection of the homozygous BRAF^V600E^ mutation in a few single cells was considered to be partly due to the failure of PCR to amplify the wild-type allele, that is, allele drop-out (ADO), which is a common problem in single-cell PCR (Piyamongkol *et al*, 2003). Although some of the wild-type sequences detected in this experiment may also be explained by the ADO of mutant alleles, it is obvious that the large number of wild-type sequences detected in melanoma tissues cannot be explained by ADO alone. As for MMG1, the ratio of 10 : 1 for wild-type BRAF and homozygous V600E mutation was significantly uneven by the two-tailed binominal test (*P*=0.012), indicating that the result is unlikely to be due to ADO (the null hypothesis here was that all the cells containing wild-type BRAF and homozygous BRAF mutation were artifacts by ADO, on the premise that the chance of ADO was the same for a mutant allele and a wild-type allele). Contamination of non-melanoma cells by non-specific binding to immunobeads was unlikely, because we had previously tested and confirmed the specificity of immunomagnetic cell isolation using anti-HMW-MAA antibodies (Lin *et al*, 2009). Thus, the results indicate that most primary melanomas are polyclonal, consisting of BRAF-mutant, as well as BRAF-wild-type tumour cells. BRAF mutation heterogeneity observed in the primary melanoma cell line MMG1 strongly supports this conclusion.

To further reveal the heterogeneity of BRAF mutations within primary melanoma, we examined 10 tumours (one melanoma on chronic sun-damaged skin, four melanomas on non-chronic sun-damaged skin, two acral melanomas and three mucosal melanomas) by the sensitive Mutector assay detecting the BRAF^V600E^ mutation (TrimGen Corporation), as well as by cloning and sequencing of separated alleles (Table 2). We obtained relatively pure tumour cell populations by manual microdissection or laser-capture microdissection with the aid of gp100 immunostaining (Supplementary Figure S1c). Conventional direct sequencing of PCR products of BRAF exon 15 revealed that three tumours harboured the V600E mutation, one tumour had the V600K mutation, and the remaining six tumours were wild type. However, the sensitivity of detecting heterozygous mutations in conventional direct sequencing is rather low, as tested in our previous study (Ichii-Nakato *et al*, 2006); the mutant peak is reliably discernable only when heterozygous mutant cells comprised >20% of samples. As expected, the more sensitive Mutector assay, which can detect as little as 5% of heterozygous BRAF^V600E^ mutation (Ichii-Nakato *et al*, 2006), showed positive results in five tumours that were wild type by direct sequencing. The result suggests that a substantial number of primary tumours, labelled as BRAF-wild type by conventional direct sequencing, actually contain a small fraction of BRAF^V600E^ cells. The tumour in case 6, which mostly consisted of cells with the BRAF^V600K^ mutation, may also contain a small fraction of BRAF^V600E^ cells, as the Mutector assay was positive. To directly demonstrate such a small fraction of mutant alleles, we cloned the PCR amplicons of BRAF exon 15 and sequenced individual alleles. A total of 40 bacterial subclones were sequenced for each sample. As expected, all the samples that showed wild type by direct sequencing but positive for the Mutector assay contained 2–7 BRAF^V600E^ mutant alleles (Supplementary Figure S1d). One BRAF^V600E^ mutant allele was detected in the tumour in case 6, while the V600K mutation prevailed in this tumour. Two primary melanomas (cases 12 and 13) had minor BRAF mutant alleles other than V600E, such as K601E and V600K. These results further confirmed the heterogeneity of BRAF mutations within primary melanomas.

To examine whether BRAF mutant alleles are selected for in melanoma progression, we enumerated BRAF mutant alleles by subcloning in three cases where the pairs of primary tumour and recurrent primary tumour or metastases were available for analyses (Table 3). RPM-10 was a recurrence of a primary tumour PM-10 that developed 3 years later. Sequencing of bacterial colonies showed an increase of BRAF^V600E^ mutant alleles in the recurrent tumour; this was reflected in the change in the results of direct sequencing from wild type to the BRAF^V600E^ mutation. In case 16, V600-K601E alleles found in the primary tumour increased in the lymph node metastasis. Case 17 was an autopsy case. The primary tumour of this case (PM-17) was wild type for BRAF by conventional sequencing. However, sequencing of as many as 80 cloned PCR amplicons revealed a small fraction of V600K mutant alleles, which were predominated in seven out of nine metastases, each obtained from different anatomical sites. These results strongly suggest the selection of BRAF mutant alleles in melanoma progression, as reported by others (Dong *et al*, 2003; Kirschner *et al*, 2005).

## Discussion

Since the discovery of frequent activating BRAF mutations in melanoma (Davies *et al*, 2002), myriads of papers have published reporting the detection of BRAF mutations in melanoma (reviewed in Hocker and Tsao, 2007). These include the landmark paper that described marked difference of BRAF mutation frequencies in the different types of melanoma; that is, ∼60% of melanomas on non-chronic sun-damaged skin had BRAF mutations, whereas the mutations were rather infrequent (11–23%) in melanomas on chronic sun-damaged skin, mucosa and acral skin (Curtin *et al*, 2005). However, most of these previous studies utilised PCR and direct sequencing in the mutation detection, which could not reliably detect heterozygous BRAF mutations when the DNA samples contained <20% of mutant cells (Ichii-Nakato *et al*, 2006; Houben *et al*, 2008). Thus, it is possible that many melanomas labelled as wild-type BRAF by direct sequencing may carry mutations, albeit at low levels below the sensitivity of the method (Greene *et al*, 2009). In this study, we employed three different methods, including single-cell mutation analysis, shifted termination assay (Mutector) and subcloning, all of which were sensitive enough to detect minor mutant alleles. We actually demonstrated a small population of melanoma cells harbouring activating BRAF mutations in a substantial number of tumours that were wild type by direct sequencing. Most of the mutations found were V600E, but several other mutations were also identified, such as V600K, V600M, V600-K601E and K601E, all of which had been reported previously in melanomas (Garnett and Marais, 2004). Thus, most primary melanoma lesions so far examined consist of melanoma cells that contained wild-type BRAF admixed with melanoma cells that contained V600E and other BRAF mutations.

*In vivo* and *in vitro* experiments have shown that activating BRAF mutations, such as V600E, stimulate constitutive cell signalling, growth factor-independent proliferation and transformation of immortalised melanocytes (Hingorani *et al*, 2003; Wellbrock *et al*, 2004; Hoeflich *et al*, 2006). Thus, it may be surprising that melanoma cells acquiring activating BRAF mutations constitute only a minor subpopulation of primary tumours and do not outgrow BRAF-wild-type cells. One possible explanation is that these cells with BRAF mutations undergo senescence, as has been demonstrated in melanocytic nevus (Michaloglou *et al*, 2005; Gray-Schopfer *et al*, 2006). However, this is unlikely because the minor population of melanoma cells with BRAF mutations became predominant in a recurrent primary tumour or metastases that developed in the same patients (Table 3). Furthermore, the expression of IGFBP7, which induces senescence in melanocytes acquiring the BRAF^V600E^ mutation, was not observed in BRAF^V600E^-positive melanoma tissues, whereas BRAF^V600E^-positive nevi expressed high levels of IGFBP7 (Wajapeyee *et al*, 2008). Another possibility is that RAS/RAF/MEK/ERK signalling is still subject to regulation in melanoma cells even in the presence of constitutively active BRAF. It has recently been shown that the phospho-ERK staining was not correlated with the mutational status of NRAS and/or BRAF in melanoma tissues, and that cultured BRAF-mutant melanoma cells downregulated RAS/RAF/MEK/ERK activation when cultured at high densities or under non-adherent conditions (Houben *et al*, 2008).

The finding of the selection of mutant BRAF alleles in melanoma progression appears to be significant in view of the recent development of selective BRAF^V600E^ kinase inhibitors (Sala *et al*, 2008; Tsai *et al*, 2008) that showed successful preliminary clinical results (Flaherty *et al*, 2010). Although BRAF mutations are thought to be rare in acral and mucosal melanomas (Curtin *et al*, 2005), primary tumours of these types of melanomas frequently contain minor populations of BRAF-mutant clones, which are likely to predominate in metastases. It is therefore crucial to genotype metastatic tumours before administrating BRAF^V600E^-selective drugs to identify patients who are likely to respond.

Finally, polyclonality of BRAF mutations in primary melanomas indicates that BRAF mutation is not a founder event in melanomagenesis. As has been recently shown in acute lymphocytic leukaemia (Greaves, 2009), it is speculated that precancerous melanocytes already harbouring an unknown first hit may subsequently acquire multiple driver mutations; thus, the acquisition of BRAF mutation might be one of these secondary events. BRAF-wild-type clones present in primary tumours and metastases are likely to contain mutations or copy number alterations affecting genes other than BRAF, such as NRAS, KIT, cyclin D1, PTEN and CDKN2A (Curtin *et al*, 2005, 2006). Future studies examining mutation profiling on a single-cell level (Greaves, 2009) would reveal a complex clonal evolution in melanoma development and progression.

## Figures and Tables

**Table 1:** Polyclonality of BRAF mutations in primary melanoma as revealed by single-cell PCR and sequencing

**Sample no.**	**Age (years)**	**Sex**	**Type of melanoma**	**No. of cells with heterozygous BRAF mutations^a^**	**No. of cells with homozygous BRAF mutations^a^**	**No. of cells with wild-type BRAF**	**Total no. of cells**
PM-1	—	F	Acral	7 (V600E)+2 (K601R)	2 (V600E)	45	56
PM-2	21	F	NCSD	14 (V600E)	3 (V600E)	31	48
PM-3	53	F	NCSD	4 (V600E)+3 (V600M)	3 (V600E)+2(V600-K601E)	40	52
PM-4	85	F	Acral	8 (V600E)	1 (V600E)	41	50
PM-5	47	F	Acral	0	0	50	50
MMG1	—	—	Acral	29 (V600E)	1 (V600E)	10	40

Abbreviations: Acral=acral melanoma; MMG1=a cell line established from primary acral melanoma; NCSD=melanoma on non-chronic sun-damaged skin; PM=primary melanoma.

a V600E, T1799A; K601R, A1802G; V600M, G1798A; V600-K601E ,TGA1799-1801del.

**Table 2:** Polyclonality of BRAF mutations in primary melanoma as revealed by the Mutector assay and subcloning

								**Subcloning^c^**	
**Sample no.**	**Age (years)**	**Sex**	**Tumour thickness (mm)**	**Type of melanoma**	**Tissue^a^**	**Direct sequencing**	**Mutector (OD ratio)^b^**	**No. of colonies with mutant BRAF**	**No. of colonies with wild-type BRAF**
PM-6	85	M	13	CSD	FFPE	V600K	Positive (8.9)	18 (V600K)+1 (V600E)	21
PM-7	57	M	4.8	NCSD	FFPE	Wild type	Positive (9.04)	3 (V600E)	37
PM-8	68	F	4.1	NCSD	FFPE	Wild type	Negative (0.53)	0	40
PM-9	29	F	4	NCSD	Frozen	V600E	Positive (32.51)	20 (V600E)	20
PM-10	32	F	0.8	NCSD	FFPE	Wild type	Positive (4.75)	2 (V600E)	38
PM-11	50	M	4.1	Acral	FFPE	Wild type	Positive (14.47)	7 (V600E)	33
PM-12	69	F	3.5	Acral	FFPE	V600E	Positive (27.34)	18 (V600E)+1 (V600K)	21
PM-13	77	F	1.5	Mucosal	Frozen	V600E	Positive (31.69)	9 (V600E)+1 (K601E)	30
PM-14	74	F	30	Mucosal	Frozen	Wild type	Positive (8.64)	2 (V600E)	38
PM-15	79	F	6	Mucosal	FFPE	Wild type	Negative (0.17)	0	40

Abbreviations: Acral=acral melanoma; CSD=melanoma on chronic sun-damaged skin; FFPE=formalin-fixed paraffin-embedded tissues; MMG1=a cell line established from primary acral melanoma; Mucosal=mucosal melanoma; NCSD=melanoma on non-chronic sun-damaged skin; PM=primary melanoma.

a All the FFPE sections were stained with gp100 protein, and pure tumour tissues were collected by laser microdissection.

b OD ratio >2 were regarded as positive (Ichii-Nakato *et al*, 2006).

c A total of 40 bacterial colonies were sequenced for each sample. V600E, T1799A; V600K, GT1798-99AA; K601E, A1801G.

**Table 3:** Selection of BRAF mutant alleles in the progression of melanoma

							**Subcloning (no. of colonies)**	
**Sample no.**	**Age (years)**	**Sex**	**Type of melanoma**	**Site**	**Tissue**	**Direct sequencing^a^**	**BRAF mutations^a^**	**Wild type**
PM-10	32	F	NCSD	Forearm	FFPE	Wild type	2(V600E)	38
RPM-10				Forearm	FFPE	V600E	7(V600E)	33
PM-16	53	F	NCSD	Neck	Frozen	V600−K601E	9(V600−K601E)+2(V600E)	30
MM-16				Cervical LN	Frozen	V600−K601E	27(V600-K601E)	13
PM-17	67	M	NCSD	Right shoulder	Frozen	Wild type	8(V600K)+1(K601E)	71
MM-17-1				Skin	Frozen	V600K	13(V600K) +1(K601E) +1(K601K)	15
MM-17-2				Right lung	Frozen	Wild type	NE	NE
MM-17-3				Small intestine	Frozen	V600K	14(V600K)	26
MM-17-4				Right chest wall	Frozen	V600K	15(V600K)+1(K601E)	54
MM-17-5				Pulmonary LN	Frozen	Wild type	NE	NE
MM-17-6				Supraclavicular LN	Frozen	V600K	9(V600K)+1(K601E)	30
MM-17-7				Infraclavicular LN	Frozen	V600K	22(V600K)	17
MM-17-8				Axillary LN	Frozen	V600K	12(V600K)	28
MM-17-9				Diaphragm	Frozen	V600K	11(V600K)	28

FFPE=formalin-fixed, paraffin-embedded tissue; LN=lymph node; MM=metastasis; NCSD=melanoma on non-chronic sun-damaged skin; NE=not examined; PM=primary melanoma; RPM=recurrent primary melanoma.

a V600E, T1799A; V600-K601E, TGA1799-1801del; V600K, GT1798-99AA; K601E, A1801G.
