# Supplementary figures and images for: Polyclonality of BRAF mutations in primary melanoma and the selection of mutant alleles during progression
Source: Br J Cancer. 2011 Jan 11;104(3):464–8. doi: 10.1038/sj.bjc.6606072 (PMC3049568; doi:10.1038/sj.bjc.6606072)

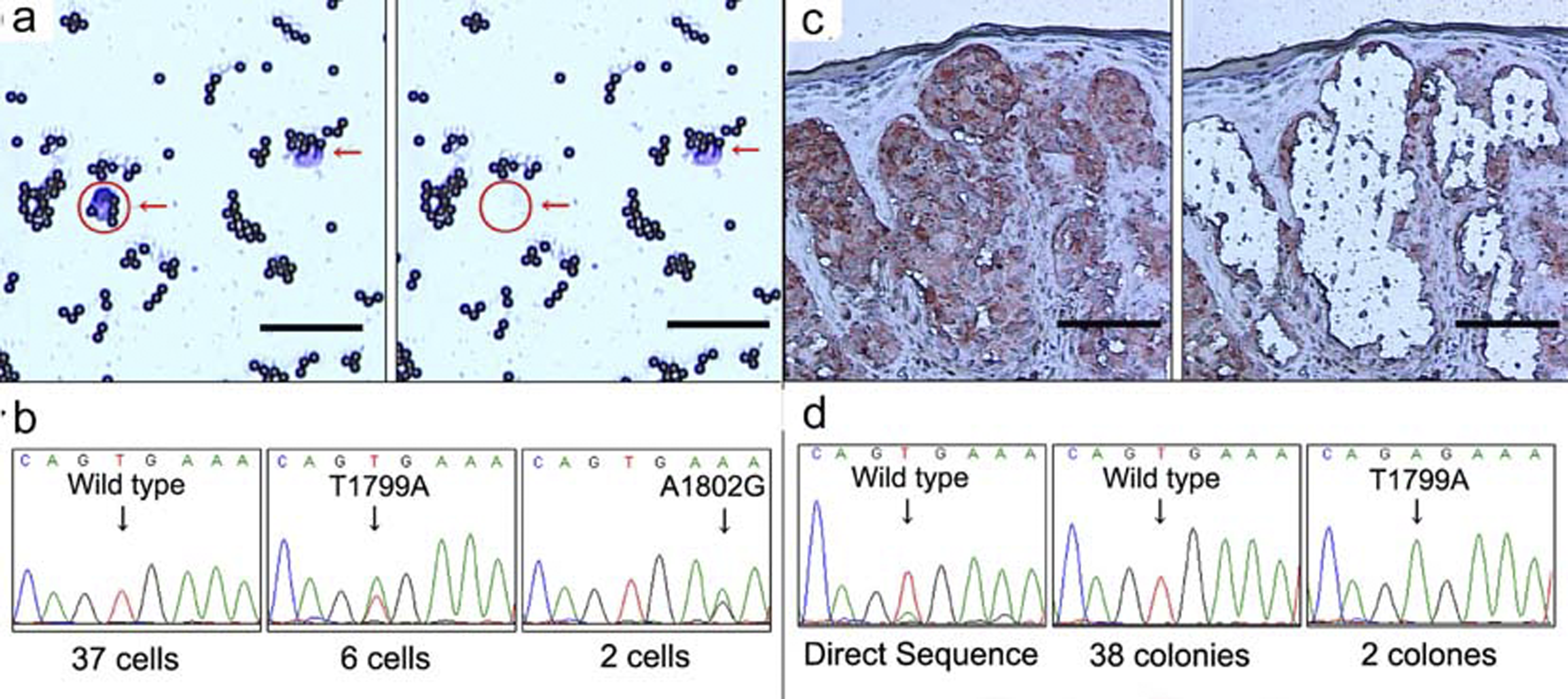

Supplement: Supplementary Figure 1 [file 6606072x1.tif]
