# Supplementary material for: Polyclonality of BRAF mutations in primary melanoma and the selection of mutant alleles during progression
Source: Br J Cancer. 2011 Jan 11;104(3):464–8. doi: 10.1038/sj.bjc.6606072 (PMC3049568; doi:10.1038/sj.bjc.6606072)
Supplement: Supplementary Figure Legend [file 6606072x2.doc]

**Figure S1 Polyclonality of BRAF mutations in primary melanoma.**

**(a)** Single melanoma cells (arrows: large purple dots) isolated from a primary melanoma tissue (PM-1) were captured by human high molecular weight melanoma associated antigen-specific monoclonal antibodies and imunomagnetic beads (small dots). One of the beads-captured melanoma cell (encircled) was procured by laser-capture microdissection. Bar= 40μm. **(b)** Sequencing charts of single melanoma cells (PM - 1) showing heterogeneity of BRAF mutations. Thirty seven cells contained wild-type BRAF, 6 cells the V600E (T1799A) mutation, and two cells the K601R (A1802G) mutation.
